# Mental Health Outcomes of Healthcare Providers During COVID-19 Pandemic in Saudi Arabia: A Cross-Sectional Study

**DOI:** 10.3389/fpubh.2021.625523

**Published:** 2021-05-28

**Authors:** Sultana A. Alhurishi, Khalid M. Almutairi, Jason M. Vinluan, Ahmad E. Aboshaiqah, Mohammed A. Marie

**Affiliations:** ^1^Department of Community Health Sciences, College of Applied Medical Sciences, King Saud University, Riyadh, Saudi Arabia; ^2^College of Nursing, King Saud University, Riyadh, Saudi Arabia; ^3^Clinical Laboratory Sciences Department, College of Applied Medical Sciences, King Saud University, Riyadh, Saudi Arabia

**Keywords:** COVID-19, mental health, healthcare providers, Saudi Arabia, pandemic

## Abstract

**Objective:** In this descriptive cross-sectional study we aimed, to assess the level of depression, anxiety, insomnia and distress symptoms experienced by healthcare providers during the COVID-19 pandemic in Saudi Arabia.

**Methods:** All healthcare providers currently working in different hospitals were invited to participate in this study. Data gathering started in March 2020 to May 2020. The participants answered a five-part questionnaire which includes demographic data, a 9-item Patient Health Questionnaire, a 7-item Generalized Anxiety Disorder, a 7-item Insomnia Severity Index, and a 22-item Impact of Event Scale-Revised, which assess the level of depression, anxiety, insomnia, and distress.

**Results:** Out of 200 healthcare providers, 40% were males. 52% were aged 31–40 years old, 61% were married. The majority of the participants were Saudi nationals (84%), 74% were nurses, 11% were physicians and 15% were other healthcare providers. More than half of the participants worked as front-liners (57%). Overall, 73, 69, 62, and 83% of all healthcare providers reported symptoms of depression, anxiety, insomnia, and distress, respectively. The analysis showed severe symptoms level of depression for physicians and nurses was 35% and 20% (*p* < 0.05), respectively. Only three of the independent variables made a unique contribution to the model (gender, profession, and working position) (*p* < 0.05).

**Conclusion:** COVID-19 pandemic has a significant impact on the mental health of healthcare providers in Saudi Arabia. Female nurses and healthcare providers working in the frontline who were directly treating patients with COVID-19 are at increased risk of severe depression, anxiety and distress.

## Introduction

The novel coronavirus disease (COVID-19) has first reported in Wuhan, Hubei province of China and demonstrated an exponential growth trend in other cities and around the World (1, 2). COVID-19 is a clinical syndrome that exhibits mild upper respiratory illness to severe pneumonia and acute respiratory distress (3, 4). The virus spread within weeks to different provinces in China and reached other 215 countries such as Italy, Spain, UK, France, and the USA. The COVID-19 outbreak was declared by the World Health Organization (WHO) declared as a pandemic (5). As of April 22, 2021, the total number of COVID-19 cases was 143,445,675 with 3,051,736 deaths worldwide (6).

Dealing with this critical condition, the government of Saudi Arabia through the Ministry of Health and other authorities has enforced restrictions on flights from and to China as an early preventive measure (7). The first case of COVID-19 in Saudi Arabia was identified on March 2, 2020, and as of April 18, 2020 cases increased to 8,200 with reported deaths of 92. Other preventive measures by the government were the suspension of classes and 14 days' isolation and quarantine in hotels for travelers who came back to the country (7). Umrah, an Islamic pilgrimage performed by thousands of Muslims in Makkah, was also suspended to contain the COVID-19 outbreak. A complete lockdown was also implemented, such as banning residents from leaving and circulating between cities and regions, including mass prayer in mosques to prevent the exportation of cases to other cities and regions (7).

With the increasing number of COVID-19 cases worldwide, particularly in Saudi Arabia, health care workers must face this highly infectious disease with a greater fatality rate. They serve as a front line directly involved to care and treat patients, resulting indirectly involved to care and treating caring and treating patients, resulting in work under tremendous pressure. These health care workers are at risk of having psychological distress and develop other mental health symptoms (8, 9). Factors such as uncertainty, an increasing number of cases and death, overwhelming workload, fear of contagion, and fear of infecting others or family members, stigmatization and discrimination, and shortage of personal protective equipment, may contribute to the psychological burden of health care workers (8, 9).

In the past, studies about infectious disease outbreaks, like the SARS epidemic, led to panic and anxiety among health care workers who were on the front lines of battle, which led to psychologic morbidity (10–13). Healthcare providers in Saudi Arabia battled multiple infectious disease outbreaks such as severe acute respiratory syndrome (SARS) in 2003 and the Middle East Respiratory Syndrome (MERS-COV) in 2012 (14, 15). Previous studies have found that healthcare workers during the MERS-COV outbreak in Saudi Arabia caused emotional distress and the main stressors were own safety and his/her family (16, 17). With the recent occurrence of COVID-19, the risk of emotional turmoil among healthcare providers during this outbreak is high (18, 19). As more COVID-19 cases have exponentially emerged in Saudi Arabia, this evolving situation is likely to put healthcare professionals at risk which may all contribute to the mental burden of healthcare providers.

Evaluation of mental health among frontline health care workers is relatively scarce. Health care workers are at risk of psychological distress by the crisis and experience. Mental health plays a vital role in managing emerging diseases and crises like the COVID-19 outbreak. To date, there is a scarcity of evidence about the mental health outcomes of healthcare providers, particularly in this region. Since majority of the literature identified in this emerging field mostly came from the Far East and Western countries. This study may also provide baseline data to formulate psychological assistance programs or interventions targeting frontline health care workers. To our knowledge, there has been little assessment related to mental health outcomes of health care workers; thus, the purpose of this study was to assess the mental health burden of health care workers during the COVID-19 outbreak in Saudi Arabia.

## Methods

### Design

A descriptive cross-sectional study.

### Study Population and Procedure

All healthcare providers currently working in different hospitals in Saudi Arabia were invited to participate in this study. As of 2020, there are 504 hospitals in Saudi Arabia which are regulated by the Ministry of Health. Ethical clearance was obtained from the Institutional Review Board of King Fahad Medical City, Riyadh, Saudi Arabia before data gathering. The data collection started in April 2020 and was completed in May 2020. A convenience sampling was used because of the current situation and data were collected using an online survey. The questionnaire link was sent to different healthcare provider through social media. In addition, Snowball technique helped to disseminate the survey link as each healthcare providers were requested to forward the questionnaire link to their colleagues.

### Instruments

All participants answered a five-part questionnaire that assesses the symptoms of depression, anxiety, insomnia, and distress. The first part of the questionnaire was demographic characteristics data that include age, gender, marital status, educational attainment, employment status. The second part of the questionnaire was the 9-item Patient Health Questionnaire (PHQ-9; range, 0–27) (20), while the 7-item Generalized Anxiety Disorder (GAD-7) scale (range, 0–21) was used to identify the anxiety level among healthcare providers (21). All scores were calculated and interpreted using a scoring manual and previous studies (20–23). For PHQ-9, a total score between 15 and 21 was considered severe depression, 0–4 (normal), 5–9 (mild), and 10–14 with moderate depression. Regarding GAD-7 anxiety, 15–21 was considered with severe anxiety, 0–4 (normal), 5–9 (mild), and 10–14 having moderate anxiety. The 7-item Insomnia Severity Index (ISI; range, 0–28) was used to assess and categorized ISI, normal (0–7), subthreshold (8–14), moderate (15–21), and severe (22–28) insomnia (22). The last part of the questionnaire was the 22-item Impact of Event Scale – Revised (IES-R; range, 0–88) and will be recorded as normal (0–8), mild (9–25), moderate (26–43), and severe (44–88) distress (23).

### Statistical Analysis

Data were entered and analyzed using SPSS windows version 22. All categorical data were presented as frequencies and percentages, while continuous data were presented as mean ± SD. Medians and interquartile ranges (IQRs) were used for not normally distributed data. Mann–Whitney *U-*test and Kruskal – Wallis test was used to examine the severity of symptoms between healthcare providers. Logistic regression was done to assess the significant predictors and outcomes. Shapiro-Wilk test was applied to check the normality of the distribution of data. *P*-value was set at < 0.05 and considered statistically significant.

## Results

Table 1 shows the demographic characteristics of the study participants. A total of 200 healthcare providers participated in the study, out of which 40% (*N* = 80) were males. More than half of the participants were aged 31–40 years (52%), were married (61%) and had an educational level of bachelor's degree (52%). The majority of the participants were Saudi nationals (84%) and worked in government (92%). Of the 200 healthcare providers, 147 (74%) were nurses, 23 (11%) were physicians and 30 (15%) were other healthcare providers. More than half of the participants worked as a frontline who directly care for suspected/positive cases of COVID-19 (58%) and 42% worked as second-line healthcare providers. Twenty-six percent of the participants worked in primary care hospitals, 24% in tertiary hospitals and 19% in the specialized medical center. A total of 82 participants (41%) lived in Riyadh, 70 participants had 5 years and below as a healthcare professional (35%) and 61% of participants had 5 years and below working in the current facility.

**Table 1:** Demographic characteristic of the participants.

**Variable**	***N* = 200**	**%**
**Age**	Mean 33.2 SD 6.4	
21–30 years	74	37
31–40 years	104	52
41 and above	22	11
**Gender**		
Male	80	40
Female	120	60
**Nationality**		
Saudi	169	84
Non-Saudi	31	16
**Marital status**		
Single	77	39
Married	123	61
**Educational level**		
Diploma	26	13
Bachelor	104	52
Post graduate	70	35
**Employment status**		
Private	16	8
Government	184	92
**Type of hospital**		
Primary	52	26
Secondary	36	18
Tertiary	49	24
Specialize medical center	38	19
Other	25	13
**Profession**		
Nurse	147	74
Physician	23	11
Others	30	15
**Working position**		
Frontline	115	58
Second-line	85	42
**Place of residence**		
Riyadh	82	41
Jeddah	18	9
Dammam	7	4
Madinah	12	6
Other	81	40
**No. of years as a healthcare professional**	Mean 9.3 SD 6.1	
5 years and below	70	35
6–10 years	49	25
11–15 years	81	40
**No. of years working in this hospital**	Mean 5.8 SD 5.1	
5 years and below	123	61
6–10 years	43	22
11–15 years	34	17

Table 2 presents the level of depression, anxiety, insomnia and distress among healthcare providers in the total cohort and by subgroups. Analysis of scores in all four scales shows the median (IQR) scores on the PHQ-9 for depression were 9.0 (4–13), 10.0 (3–13) the GAD-7 for anxiety, 8.0 (5–13) the ISI for insomnia, and 31.0 (13–44) the IES-R for distress for all healthcare providers. Overall, 73, 69, 62, and 83% of all healthcare providers reported symptoms of depression, anxiety, insomnia, and distress, respectively. The analysis shows that severe symptoms level of depression for physicians and nurses were 35 and 20% (*p* < 0.05), respectively. Female participants reported severe symptoms level of depression [*N* = 26 (21.7) vs. *N* = 12 (15%), *P* < 0.05] and anxiety [*N* = 21 (17) vs. *N* = 9 (11%), *P* < 0.05] than males. A considerable proportion of participants had severe symptoms of distress among physicians (*N* = 8, 35%) and nurses (*N* = 41, 28%) (*P* < 0.05). Low to the absence of severe symptoms of insomnia were found among healthcare providers who participated in the study. There were no differences in the working position of healthcare providers for scores of depression, anxiety, insomnia and distress.

**Table 2:** Level of depression, anxiety, insomnia and distress among healthcare providers in total cohort and subgroups.

		**Profession**				**Sex**			**Working position**		
**Severity category**	**Total score, Median, (IQR)/No. of total cases (%)**	**Nurse**	**Physician**	**Others**	***P*-value**	**Male**	**Female**	***P*-value**	**Frontline**	**Second-line**	**P-value**
**PHQ-9, depression symptoms**	9 (4–13)										
None-minimal (0–4)	53 (26.5)	38 (25.9)	5 (21.7)	10 (33.3)	**0.043**	20 (25)	33 (27.5)	**0.028**	24 (20.9)	29 (34.1)	0.250
Mild (5–9)	52 (26)	38 (25.9)	7 (30.4)	7 (23.3)		20 (25)	32 (26.7)		33 (28.7)	19 (22.4)	
Moderate (10–14)	57 (28.5)	42 (28.6)	3 (13)	12 (40)		28 (35)	29 (24.2)		37 (32.2)	20 (23.5)	
Severe (15–21)	38 (19)	29 (19.7)	8 (34.7)	1 (3.3)		12 (15)	26 (21.7)		21 (18.3)	17 (20)	
**GAD-7 anxiety**	10 (3–13)										
Normal	62 (31)	50 (34)	3 (13)	9 (30)	0.209	23 (28.7)	39 (32.5)	**0.038**	31 (27)	31 (36.5)	0.182
Mild	71 (35.5)	47 (32)	10 (43.5)	14 (46.7)		30 (37.5)	41 (34.2)		43 (37.4)	28 (32.9)	
Moderate	37 (18.5)	30 (20.4)	4 (17.4)	3 (10)		18 (22.5)	19 (15.8)		26 (22.6)	11 (12.9)	
Severe	30 (15)	20 (13.6)	6 (26.1)	4 (13)		9 (11.3)	21 (17.5)		15 (13)	15 (17.6)	
**Insomnia symptoms**	8 (5–13)										
Absence	75 (37.5)	53 (36.1)	6 (26.1)	16 (53.3)	0.240	26 (32.5)	49 (40.8)	0.211	40 (34.8)	35 (41.2)	0.626
Subthreshold	86 (43)	67 (45.6)	9 (39.1)	10 (33.3)		36 (45)	50 (41.7)		52 (45.2)	34 (40)	
Moderate	36 (18)	25 (17)	7 (30.4)	4 (13.3)		18 (22.5)	18 (15)		22 (19.1)	14 (16.5)	
Severe	3 (1.5)	2 (1.4)	1 (4.3)	0		0	3 (2.5)		1 (0.9)	2 (2.4)	
**IES-R, distress symptoms**	31 (13–44)										
Normal	34 (17)	23 (15.6)	3 (13)	8 (26.7)	**0.012**	14 (17.5)	20 (16.7)	0.558	19 (16.5)	15 (17.6)	0.712
Mild	70 (35)	53 (36.1)	6 (26.1)	11 (36.7)		25 (31.3)	45 (37.5)		38 (33.0)	32 (37.6)	
Moderate	43 (21.5)	30 (20.4)	6 (26.1)	7 (23.3)		21 (26.3)	22 (18.3)		28 (24.3)	15 (17.6)	
Severe	53 (26.5)	41 (27.9)	8 (34.8)	4 (13.3)		20 (25)	33 (27.5)		30 (26.1)	23 (27.1)	

*GAD-7, 7-item generalized anxiety disorder; IES-R, 22-item impact of event scale–revised; ISI, 7-item insomnia severity index; PHQ-9, 9-item patient health questionnaire. P-value significant at P < 0.05. Bold values are considered significant (P-value significant at P < 0.05)*.

Logistic regression was performed to assess the impact of some factors associated with healthcare providers' mental health outcomes. The model contained eleven independent variables (age, gender, nationality, marital status, educational level, type of hospital, profession, working position, place of residence, number of years as health care professional and number of years working in the hospital). As shown in Table 3, only three independent variables made a unique contribution to the model (gender, profession, and working position). Gender was associated with severe symptoms of depression and anxiety. Female respondents had two times more likely to report or have depression (OR, 1.94; 95% CI, 0.89–4.22; *P* = < 0.05) and anxiety symptoms (OR, 1.42; 95% CI, 0.34–5.94; *P* = < 0.05) than males. Compared with the profession, working as a nurse was associated with more severe symptoms of anxiety than physicians and other healthcare providers (OR, 0.57; 95% CI, 0.32–1.30; *P* < 0.05), (OR, 0.72; 95% CI, 0.05–9.31; *P* < 0.05). In addition, working as physician was associated with more severe symptoms of depression (OR, 2.43; 95% CI, 0.91–6.47; *P* = 0.50), insomnia (OR, 2.32; 95% CI, 0.89–6.07; *P* = < 0.05), and distress (OR, 1.44; 95% CI, 0.56–3.68; *P* < 0.05). With regards to working position, the odds ratio of 0.48 and 0.92 was <1, indicating that working in the frontline who were directly treating patients with COVID-19 appeared to be a significant risk factor for severity of depression (OR, 0.48; 95% CI, 0.09–2.37; *P* = < 0.05) and distress (OR, 0.92; 95% CI, 0.44–1.92; *P* = < 0.05).

**Table 3:** Predictors associated for mental health outcomes identified by multivariable logistic regression analysis.

	**PHQ-9, depression symptoms**		**GAD-7 anxiety**		**Insomnia symptoms**		**IES-R, distress symptoms**	
	**OR (95%CI)**	***P*-value**	**OR (95%CI)**	***P*-value**	**OR (95%CI)**	***P*-value**	**OR (95%CI)**	***P*-value**
**Age**		0.645		0.450		0.732		0.523
21–30 years	1 [Reference]		1 [Reference]		1 [Reference]		1 [Reference]	
31–40 years	1.11 (0.33–3.70)		0.93 (0.37–2.36)		1.57 (0.50–4.90)		1.05 (0.35–3.10)	
41 and above	2.74 (0.30–24.72)		2.67 (0.43–16.53)		1.57 (0.17–14.53)		0.36 (0.05–2.69)	
**Gender**		**0.020**		**0.039**		0.566		0.470
Male	1 [Reference]		1 [Reference]		1 [Reference]		1 [Reference]	
Female	1.94 (0.89–4.22)		1.42 (0.34–5.94)		0.79 (0.38–1.63)		1.27(0.65–2.46)	
**Nationality**		0.077		0.891		0.161		0.119
Saudi	1 [Reference]		1 [Reference]		1 [Reference]		1[Reference]	
Non–Saudi	3.07 (0.88–10.65)		1.07 (0.38–2.96)		2.31 (0.71–7.52)		2.32 (0.80–6.75)	
**Marital Status**		0.842		0.597		0.291		0.225
Single	1[Reference]		1 [Reference]		1[Reference]		1 [Reference]	
Married	0.90 (0.33–2.46)		0.81 (0.37–1.76)		0.59 (0.22–1.55)		0.58 (0.24–1.38)	
**Educational level**		0.061		0.064		0.681		0.051
Diploma	1 [Reference]		1 [Reference]		1 [Reference]		1 [Reference]	
Bachelor	0.31 (0.09–1.05)		0.98 (0.35–2.71)		0.78 (0.21–2.90)		0.50 (0.16–1.54)	
Post graduate	0.08 (0.01–0.50)		0.33 (0.09–1.15)		0.52 (0.11–2.49)		0.17 (0.04–0.72)	
**Type of hospital**		0.199		0.339		0.390		0.366
Private	1 [Reference]		1 [Reference]		1 [Reference]		1 [Reference]	
Government	0.38 (0.08–1.67)		1.33 (0.47–3.76)		1.62 (0.53–4.93)		0.56 (0.16–1.94)	
**Profession**		**0.006**		**0.028**		**0.010**		**0.010**
Nurse	1[Reference]		1 [Reference]		1 [Reference]		1 [Reference]	
Physician	2.43 (0.91–6.47)		0.57 (0.32–1.30)		2.32 (0.89–6.07)		1.44 (0.56–3.68)	
Others	0.10 (0.13–0.81)		0.72 (0.05–9.31)		0.69 (0.13–2.26)		0.34 (0.10–1.08)	
**Working position**		**0.026**		0.372		0.884		**0.044**
Frontline	1 [Reference]		1 [Reference]		1 [Reference]		1 [Reference]	
Second–line	0.48 (0.09–2.37)		1.56 (0.73–3.31)		1.05 (0.50–2.22)		0.92 (0.44–1.92)	
**Place of residence**		0.531		0.618		0.980		0.537
Riyadh	1 [Reference]		1 [Reference]		1 [Reference]		1 [Reference]	
Other	1.43 (0.46–4.42)		0.76 (0.26–2.18)		1.01 (0.33–3.04)		0.49 (0.05–4.65)	
**No. of years as a healthcare professional**		0.093		0.272		0.929		0.168
5 years and below	1 [Reference]		1 [Reference]		1 [Reference]		1 [Reference]	
6–10 years	1.63 (0.42–6.23)		0.50 (0.17–1.48)		0.76 (0.20–2.87)		0.30 (0.08–1.10)	
11–15 years	0.62 (0.13–2.94)		0.82 (0.26–2.60)		1.12 (0.30–4.24)		1.06 (0.30–3.72)	
**No. of years working in this hospital**		0.388		0.961		0.484		0.215
5 years and below	1 [Reference]		1 [Reference]		1 [Reference]		1 [Reference]	
6–10 years	0.54 (0.14–2.01)		1.11 (0.39–3.10)		0.51 (0.63–0.16)		0.32 (0.08–1.28)	
11 and above	1.61 (0.30–8.57)		1.32 (0.38–4.51)		2.39 (0.58–9.84)		0.99 (0.25–3.85)	

*GAD-7, 7-item generalized anxiety disorder; IES-R, 22-item impact of event scale–revised; ISI, 7-item insomnia severity index; PHQ-9, 9-item patient health questionnaire. P-value significant at P < 0.05. Bold values are considered significant (P-value significant at P < 0.05)*.

## Discussion

This descriptive cross-sectional study provides insight into the mental health status of healthcare providers during COVID-19 outbreak in Saudi Arabia. The main findings of the present study indicate that a considerable proportion of healthcare providers who worked during the COVID-19 outbreak reported symptoms of severe depression, anxiety, insomnia, and distress. These results are similar to the findings among healthcare workers exposed to coronavirus disease in Wuhan, China, demonstrating that healthcare workers experienced depression, anxiety, insomnia, and distress during the outbreak (18). Several studies conducted in Saudi Arabia have shown similar findings regarding the negative emotional status among health workers during the COVID-19 pandemic (24–27). For example (24), have assessed the mental health outcomes in Saudi Arabia and have found that the majority have mild to low symptoms (20). The lower proportion of health care workers who suffer from mental issues might be explained by the high proportion of second-line health care workers in the sample (24). Meanwhile, in a cross-sectional study that assess psychological disturbances among healthcare workers in Saudi Arabia and Egypt found out that majority of the participants had depression, more than half of them had anxiety and stress, and almost 40% had inadequate sleeping (<6 h/day) (26). The difference between the level of psychological distress among healthcare providers in this region may be because of the timing of the survey which is very important in order to highlight the level of anxiety and stress within the healthcare providers. However, the findings of this study emphasize the impact of COVID-19 on the mental health of healthcare providers in Saudi Arabia.

Another highlight of this study is the factors associated with the mental health outcomes of health care providers, the study suggests that females, nurses and working in the frontline who were directly treating patients with COVID-19 were associated with experiencing severe depression, anxiety, and distress. Similarly, data from a large-scale stratified study collected in Wuhan involving 1,257 healthcare workers, described higher anxiety scale scores among women than men (19). In addition, frontline healthcare workers engaged direct care of patients were significantly associated with higher symptoms of psychological distress (depression, anxiety and insomnia) (19). In contrast, preliminary data in Jordan indicated that being male, married, age 40 and above and have more clinical experience are associated with the psychological distress of healthcare providers (28). These factors have also been linked during the previous outbreak (SARS) in which high levels of depressive symptoms were observed among healthcare workers who closely treat patients (12). From these findings, it is suggested that the psychological well-being of healthcare providers involved in acute COVID-19 outbreak appeared to be the main targets of psychiatric assessment and care. Healthcare providers working at hospitals treating patients with infectious diseases (e.g., SARS, MERS-CoV, COVID-19) outbreaks have a higher increase risk of developing or suffering from clinically significant symptoms such as depression, anxiety and distress.

In general, little is known about healthcare providers' emotional burden or mental health problem healthcare providers' emotional burden or mental health problems during the COVID-19 outbreak, particularly in Saudi Arabia. Health care providers may exceed individual coping skills. Determining the level of mental health problems of healthcare providers during the COVID-19 crisis may help develop psychological assistance programs or interventions targeting frontline health care workers during and after the COVID-19 crisis. Assessing the mental health of healthcare providers during this outbreak is essential, which will offer the pooled estimation of the level of depression, anxiety, insomnia and distress experienced by the healthcare providers.

The authors acknowledge several limitations. The generalizability of the study findings to health workers in Saudi Arabia and underrepresented regions is limited. The study has examined the emotional status using self-reported instruments only no other diagnostic tests or follow-up were used. Additionally, data collection was carried out during the second and third months when the COVID 19 pandemic started, a complete lock-down was implemented and thus, an extra burden on healthcare providers both at work and home could have existed. The sample size was not large enough, which cannot generalize the mental health outcomes of healthcare providers in Saudi Arabia although the sample size is not very high to claim generalizability, the sample has included male, female health workers with various age range etc. thus, enhanced emotional status measurements and transferability of the findings. Evidence on this study relies on small sample size, more evidence on this subject is needed.

Because of the challenging situation, the progression of mental health symptoms or psychological manifestation among healthcare providers could increase. Although the sample size of this study was small, the healthcare providers were located from significant regions and in the most affected areas of Saudi Arabia. Findings from this study might inform the current mental health support services for health practitioners. A prospective longitudinal survey is recommended to determine the impact and psychological implications among healthcare providers. These findings may help future researchers and in the clinical field as a baseline of depression, anxiety, insomnia, and distress in Saudi Arabia. This study might also help strengthen healthcare systems' capacity and identify strengthen healthcare systems' capacity and identify the potential negative impact on the public health of the COVID-19 outbreak. The Ministry of Health has established a hotline to support well-being of health care workers. Specialized clinics were established to support the mental health of employees. In addition, the Saudi Commission for Health Specialties has launched DA'EM a 24-h web-based wellness program to enhance mental health among health care workers in Saudi Arabia (29). This study could also inform the current interventions to support the mental health of health care workers in the Kingdom.

In conclusion, the COVID-19 outbreak has a significant impact on the mental health of healthcare providers in Saudi Arabia. A considerable proportion of healthcare providers reported symptoms of depression, anxiety, insomnia and depression. Female, nurses and healthcare providers working in the frontline who were directly treating patients with COVID-19 is at increased risk of severe depression, anxiety and distress. An extensive rapid psychological intervention to promote mental well-being targeting this population needs to be implemented.

## Data Availability Statement

The datasets supporting the conclusions of this article are available at the Department of Community Health Sciences, College of Applied Medical Science, King Saud University from the corresponding author on reasonable request.

## Ethics Statement

The studies involving human participants were reviewed and approved by Institutional Review Board of King Fahad Medical City, Riyadh, Saudi Arabia. The patients/participants provided their written informed consent to participate in this study.

## Author Contributions

SA and KA: conceptualization, writing—original draft, facilitate data gathering, and data analysis. JV: writing the original draft, data analysis, and facilitate data gathering. AA and MM: facilitate data gathering and writing the original draft. All authors contributed to the article and approved the submitted version.

## Conflict of Interest

The authors declare that the research was conducted in the absence of any commercial or financial relationships that could be construed as a potential conflict of interest.
